# Audio-Visual Detection Benefits in the Rat

**DOI:** 10.1371/journal.pone.0045677

**Published:** 2012-09-18

**Authors:** Stephanie Gleiss, Christoph Kayser

**Affiliations:** 1 Max Planck Institute for Biological Cybernetics, Tübingen, Germany; 2 Bernstein Center for Computational Neuroscience, Tübingen, Germany; Centre national de la recherche scientifique, France

## Abstract

Human psychophysical studies have described multisensory perceptual benefits such as enhanced detection rates and faster reaction times in great detail. However, the neural circuits and mechanism underlying multisensory integration remain difficult to study in the primate brain. While rodents offer the advantage of a range of experimental methodologies to study the neural basis of multisensory processing, rodent studies are still limited due to the small number of available multisensory protocols. We here demonstrate the feasibility of an audio-visual stimulus detection task for rats, in which the animals detect lateralized uni- and multi-sensory stimuli in a two-response forced choice paradigm. We show that animals reliably learn and perform this task. Reaction times were significantly faster and behavioral performance levels higher in multisensory compared to unisensory conditions. This benefit was strongest for dim visual targets, in agreement with classical patterns of multisensory integration, and was specific to task-informative sounds, while uninformative sounds speeded reaction times with little costs for detection performance. Importantly, multisensory benefits for stimulus detection and reaction times appeared at different levels of task proficiency and training experience, suggesting distinct mechanisms inducing these two multisensory benefits. Our results demonstrate behavioral multisensory enhancement in rats in analogy to behavioral patterns known from other species, such as humans. In addition, our paradigm enriches the set of behavioral tasks on which future studies can rely, for example to combine behavioral measurements with imaging or pharmacological studies in the behaving animal or to study changes of integration properties in disease models.

## Introduction

Multisensory information derived from our different senses provides unique behavioral benefits. These include faster reactions and better detection rates in multisensory compared to unisensory conditions [1], [2], the more rapid accumulation of information in time [3], or the facilitation of higher-level object processing [4]. The brain networks underlying these multisensory benefits have received much interest in the last decade [5], [6], [7], [8]. Pioneering work was advanced in the cat [9], [10] and studies on the primate brain have provided key insights about the computational principles and neural mechanisms underlying multisensory convergence and integration [11], [12], [13], [14]. While this body of work provides us with a growing understanding of the mechanisms and perceptual constraints underlying multisensory integration [3], there still remain many challenges for studies to directly link multisensory perception and specific neural circuits in the primate brain [13].

The development of rodent models for cognition together with the advent of tools for high-density imaging and manipulation of brain activity in behaving animals [15], [16] highlight the potential to overcome this gap in the rodent. For example, Iurilli and colleagues [17] recently combined opto-genetics, single-cell recordings and behavioral tests in mice to demonstrate that direct anatomical connections between early sensory cortices implement a cross-modal gain control that shapes the impact of sensory stimuli on perception. While studies such as this demonstrate the power of rodent models in elucidating the neural mechanisms of multisensory processing, one important constraint for rodent work remains the small number of multisensory behavioral tasks. Specifically, to link neural mechanism to perception and ultimately to the human brain, behavioral protocols are required in which rodents exhibit similar behavioral benefits as humans in comparable tasks. In previous work, for example, Sakata et al. provided evidence that rats can exhibit faster detection of audio-visual compared to auditory targets similar to humans [18] (see also [19]), and Raposo et al. devised a task in which both rats and humans exhibit similar multisensory benefits when accumulating information over time [20]. Also multisensory object discrimination tasks requiring rats to combine visual and olfactory cues have been implemented [21]. However, further behavioral protocols are needed to provide future research with a suitable collection of tasks where rodents exhibit similar behavioral benefits as known for humans.

We developed a two-response forced-choice task requiring rats to detect lateralized audio-visual targets of varying intensity. The design of this task was motivated by a body of human psychophysical literature, showing that a simultaneously presented sound can enhance the detection of visual targets [22], [23], [24], [25], [26] and the perceived luminance of light [27], even when the sound itself is not informative about the visual task [26]. Rats reliably and rapidly learned this task. Importantly, performing psychophysical tests on the animals we found that they exhibit enhanced performance rates and faster reaction times for dim visual targets when these are accompanied by a simultaneous sound. The animals' behavior in this task hence follows the classical principle that multisensory perceptual benefits are strongest when the unisensory stimuli are weakly effective in eliciting a robust behavioral response [5], [28].

## Materials and Methods

### Animals

Eight adult male rats (Long Evans; Charles River Laboratories; 4–6 weeks age at beginning of training) were used for this study. The behavioral procedures required for these experiments were approved by the local authorities (Regierungspräsidium Tübingen) and were in accordance with the guidelines of the European Community for the care and use of laboratory animals. Animals were socially group-housed in enriched environments (partly in ‘Double-Decker’ two-level cages, Tecniplast S.p.a., Italy), were maintained under an inverted 12 h dark-light cycle and were under regular veterinary inspection.

### Training apparatus and sensory stimulus presentation

**Figure 1:**
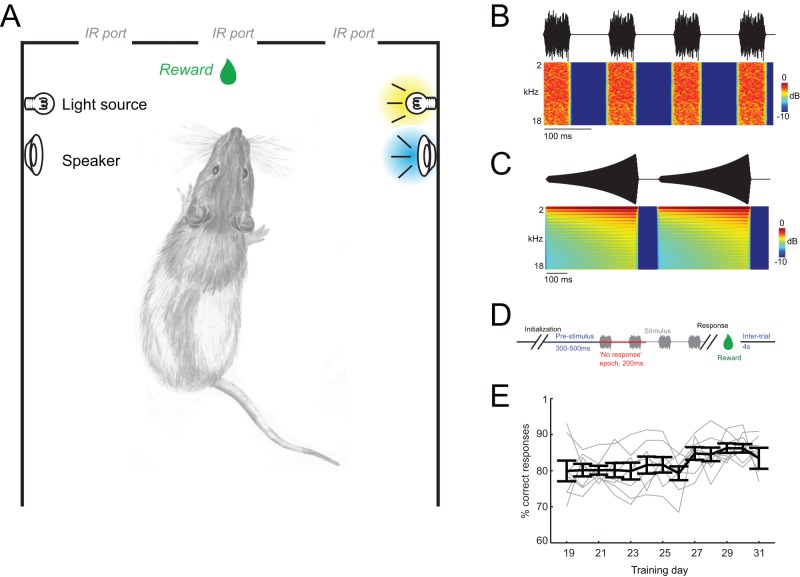
Training setup and stimuli. A) Schematic of the training setup indicating three infra-red sensitive nose-pokes, a tube for reward-delivery and the lateralized visual and acoustic stimuli. Stimuli were presented on either side and in one of three modality conditions (visual only, auditory only, audio-visual). B) Sound wave (upper) and time-frequency representation (lower panel) for the auditory white-noise stimulus. Noise pulses lasted 60 ms with 80 ms inter-pulse intervals. C) Sound wave and time-frequency representation for auditory looming sounds. Individual looming sweeps lasted 500 ms and were separated by 100 ms intervals. D) Schematic of trial timing. Trial initialization by the animal was followed by a pre-stimulus period (300–500 ms) subsequent to which the stimulus appeared on either side. The first 200 ms of stimulus presentation were defined as no-response epoch during which responses were ‘punished’ by time-outs. E) Behavioral performance (% correct responses) for the first days during the final training stage (Step 6). The animals reached stable performance above 80% in few days of training on this step. Days are indicated relative to the total training time (including all steps).

Behavioral training was performed in a custom-built operant ‘training box’ (32×25 cm wide and 45 cm high) with side walls consisting of thin aluminum bars to avoid echoes. The box itself was placed in an anechoic chamber padded with sound-attenuating foam (ambient noise level of about 40 db(A-weighted)). Three infra-red sensitive nose-poke ports (26 mm diameter; removable) were located at the front wall (20 mm spaced) and a tube for delivering liquid rewards was installed below the center nose-poke (Fig. 1A). Rewards consisted of 75 µl drops of chocolate milk mixed with baby nutrient and were delivered by a computer-controlled tubing pump (REGLO digital, Ismatec, Germany). An infrared camera for online observation of the animals and a house light were installed above the box. For stimulus presentation small head-phone speakers (5 mm diameter) and a small plastic lens (2 mm diameter) connected to a fiber-optic light guide were positioned at head level near the front wall. They were positioned such that they were at an optimal position relative to the animals head during nose-poking. Stimulus presentation, detection of nose-poking and reward delivery were controlled using custom-written behavioral protocols running in Matlab (Mathworks Inc.) on a personal computer.

Auditory and visual stimuli consisted of the lateralized presentation of noises and lights. Specifically, auditory stimuli consisted of white noise pulses (60 ms duration, 80 ms inter-pulse interval, frequency range 2–18 kHz) and were presented at 80 dB(A) SPL and on either side (Fig. 1B). Stimulus intensity and speaker transfer function were calibrated using a condenser microphone (Bruel&Kjær 4188) and 2238 Mediator sound level meter (Bruel&Kjær). Sounds were cosine on-off ramped (8 ms ramp). Visual stimuli consisted of the illumination of a white plastic lens by a fiber-optic light guide connected to a dimmable light source. Light intensity was systematically varied in six steps (1, 0.5, 0.12, 0.06, 0.03, 0.015 [cd/m^2^]) as calibrated using a Minolta Chroma Meter CS-100 (Konica Minolta, Japan). Stimuli were presented as either light only (visual condition), sound only (auditory condition) or as multisensory pair (audio-visual condition). Stimuli were presented for periods of several seconds and inter-leaved with inter-stimulus periods of variable duration (see below).

In additional control experiments we also employed i) auditory white noise bursts at softer intensity (65 dB(A) SPL); ii) an auditory stimulus that was not lateralized (not informative about the side of stimulus presentation) and which consisted of the simultaneous presentation of the white noise bursts on both speakers; and iii) we replaced lateralized white noise bursts by lateralized looming sounds. Looming sounds are complex tones of great behavioral relevance [29]. They were constructed from a 400 Hz triangular waveform rising exponentially in intensity from 0% to 100% in 500 ms (Fig. 1C). Individual looming sounds were repeated (100 ms silent) intervals to obtain a longer acoustic stimulation period as in previous behavioral studies using such sounds [30].

### Training procedure

Training was performed using standard operant conditioning with milk rewards as positive and time-outs as negative reinforces. During the training period the animals were on a food-restricted diet (ad libitum water) and their food and water intake and their weight were monitored. Controlled quantities of rat chow were provided subsequent to the daily training session. Training on the sensory discrimination task proceeded in several steps. Initially, (step 1) the animals were habituated to the training box (free exploration with random rewards, 20–60 min each, 1 day). Subsequently (step 2) they learned to collect rewards by nose poking into the center port (60 min per day, until animals reliably acquired >100 rewards per day). In the next step (step 3) we introduced the sensory stimuli. Stimuli were presented as audio-visual pairs and during each session stimuli appeared on only one side (only the nose-poke on that side was available; sides/stimuli alternated on a daily basis). Animals could collect rewards by nose poking during stimulus presentation (10 s periods) and time-out periods (indicated by activating the house light for 10 s) were triggered by nose poking during inter-stimulus periods (lasting 6 s). Training on this step proceeded until the animals reached a criterion of at least 120 correct responses per session. For this and all subsequent steps daily training was done for 60 min or maximal 260 trials per day. Subsequently, individual modality conditions were introduced (visual only, auditory only, audio-visual; step 4). Conditions were presented in pseudo-random order and training proceeded until a criterion of 80% correct stimulus detection in each modality was reached. In step 5 the actual discrimination was introduced. Stimuli on both sides were presented in pseudo-random sequence (in either modality condition) and the animals were required to respond on the correct side during stimulus presentation (10 s period; 4 s inter-stimulus; 10 s time-out following wrong response). Training proceeded until the animals reached a criterion of at least 80% correct responses in two consecutive sessions. In the final step (step 6) we changed the computer controlled stimulus timing to a self-paced protocol. The animals learned to activate the presentation of sensory stimuli by nose-poking in the center port (with a 300–500 ms delay from nose-poking to stimulus presentation). In addition, we introduced a no-response epoch (first 200 ms of stimulus presentation) during which responses triggered time-outs (Fig. 1D). The time delay and no-response epoch were introduced to overcome reflexive and often wrong responses immediately after trial initialization observed in initial tests. Such time-delays may also be required when using this paradigm during electrophysiological, pharmacological or microstimulation studies. Otherwise stimuli and timing were as before. Training continued until the animals showed a stable performance of above 80% correct responses. During all training steps stimuli were presented at a single intensity (auditory: 80 dB(A) SPL, visual: 1 cd/m^2^).

### Behavioral data collection and analysis

Psychophysical tests probing behavioral performance (% correct responses) and reaction times as a function of stimulus intensity and modality combination were performed subsequent to the above behavioral training sequence and after the animals reached stable performance. Days of behavioral data collection (including low-intensity stimuli with possibly low performance levels) were alternated with days of behavioral training (using only high intensity stimuli as during training) to maintain overall stable performance and high motivation. During psychophysical testing sessions stimuli were presented on either side (left; right), as either modality combination (auditory; visual; audio-visual), and using six intensities of the visual stimuli (keeping the auditory stimulus intensity constant). All conditions were presented in pseudo-random order and on each day we collected data on 260 trials (or for a maximum of 60 minutes).

Four different psychophysical tests were performed (in the order listed below) that differed in the nature of the acoustic stimulus. 1) Using lateralized auditory white noise bursts of 80 dB(A) intensity (frequency range 2–18 kHz). 2) Using lateralized auditory white noise bursts of 65 dB(A) intensity. 3) Using non-lateralized white noise bursts. 4) Using looming sounds instead of white noise. For each test we collected behavioral data during 8 sessions, resulting in comparable number of trials per conditions across tests. Data for different tests were acquired at different stages after the initial training procedure: Test 1 was performed after 10 and 25 days of training on the full task. Tests 2–4 were performed after the 25 day training period and in the listed order. Tests 1 and 2 were performed on all 8 animals, while tests 3 and 4 were performed on a group of four animals.

Behavioral data were collected in Matlab in form of log files produced by the behavioral control system and were subsequently analyzed using custom-written Matlab scripts. From the log files we extracted the following response types: a correct response to a stimulus (hit; nose-poke on the side of stimulus presentation); a wrong response to a stimulus (wrong; nose-poke on the other side); a response after trial initiation but before stimulus presentation or during the 200 ms no response epoch (early response); absence of a response during the self-initiated 6 s stimulus period following stimulus initialization (no response). Performance levels were calculated as the percentage of correct responses (within hit and wrong responses). Reaction times were extracted relative to stimulus onset and included the 200 ms no-response epoch. For statistical comparisons between conditions (e.g. visual vs. audio-visual at fixed intensity) we performed ANOVAs using animals as random and conditions as fixed factor and using the average performance (or reaction time) from each of the 8 testing sessions as repeated values.

## Results and Discussion

### Task acquisition

Rats were trained on a two-response forced-choice audio-visual detection task using multiple training steps. Initial stages involved the acquisition of behavioral responses (nose-poking) and the eliciting of such responses specific to the sensory stimuli. In the final task (step 6 of the training schedule, see above) rats reliably discriminated lateralized stimuli appearing in either the visual, auditory or both (audio-visual) modalities and of variable intensity. Several key features of this task are worth noting. First, the animals self-initiated the stimulus presentation, which is known to facilitate rodent behavior [31], [32]. Second, the task included a random delay between initialization and stimulus presentation, as possibly required for subsequent electrophysiological or stimulation studies (Fig. 1D). And third, it was designed as two-response forced choice task (in contrast to e.g. a Go/No-Go paradigm), which allows better control over behavioral criterion shifts and the assessment of false responses [33]. It took the animals on average 18 training days to reach the final training stage (training step 6) and they reached stable performance levels (two consecutive days above 80% correct) after total of 22±1.5 days of training (mean ± s.e.m.; Fig. 1E). Between animal variability also decreased with proloned training, as visible in Fig. 1E. The animals performed the task reliably, and with few trials on which they responded too early during the imposed no-response epoch (12.7±1% of trials) or on which the animals initiated a stimulus presentation but did not make the discriminatory response (0.5±0.1% of trials).

### Psychophysical test with an informative sound

We probed psychophysical performance levels after 10 and 25 days of training on the final task (28 and 43 days in total). In general we found that the animals displayed improved performance levels and after 25 days also faster reaction times in the audio-visual compared to visual condition, especially at low visual intensities.

**Figure 2:**
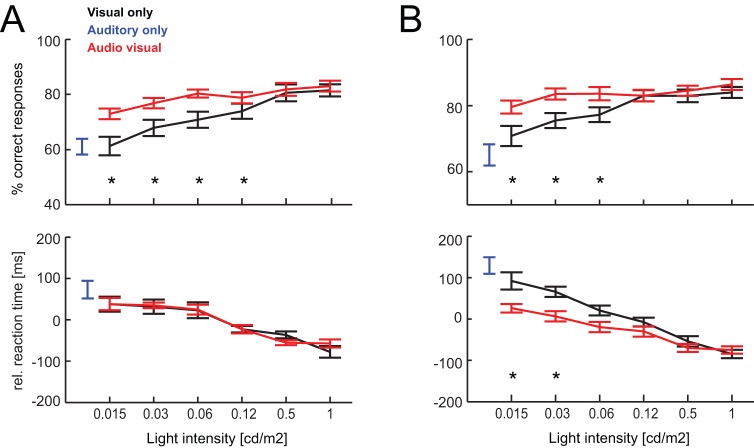
Behavioral performance – main paradigm. Behavioral performance (% correct responses, upper panel) and reaction times (lower panel) for each condition and as function of visual target intensity. Error-bars denote the mean and s.e.m. across animals (n = 8). Reaction times were normalized by subtracting each animals mean reaction time (computed across all conditions). Stars indicate statistical significance (ANOVA p<0.05). The data in A) were obtained after 10 days of training, those in B) after 25 days. Note that reaction times (e.g. for the visual condition) in B appear slower compared to A because of the normalization. Actual values for the visual conditions were comparable while those for the audio-visual conditions were shortened in B (c.f. data presented in the main text).

Psychophysical testing of all 8 animals after 10 days of training (8 testing sessions, 143±6 valid trails per condition) revealed that performance levels in the visual-only condition systematically increased with stimulus intensity, from 61±3% correct at 0.015 cd/m^2^ to 82±2% at 1 cd/m^2^ (Fig. 2A upper panel). Performance for the auditory condition was low and comparable to that for the lowest visual intensity (60±2%, F(127,1) = 0.01, p = 0.9). Importantly, discrimination performance for audio-visual stimuli was higher than for either visual or auditory stimuli for all intensities. This multisensory performance benefit was highest at the lowest visual intensity (visual 61±3% vs. audio-visual 73±2%, F(127,1) = 15.3, p<0.01) and was significant for the four lowest intensities (at least p<0.05, Fig. 2A). Performance levels for brighter visual stimuli did not differ between visual and audio-visual conditions (e.g. at highest intensity: 82±2% vs. 83±2%, p = 0.28).

Reaction times (RTs) measured during the same testing sessions also varied with target intensity. Reaction times were slower at low target intensities (0.015 cd/m^2^: 615±40 ms) and faster when the target was brighter (1 cd/m^2^: 500±20 ms) resulting a significant reaction time decrease with increasing stimulus intensity (lowest vs. highest intensity: F(127,1) = 17, p<0.01). RTs for auditory stimuli were comparable to those for the lowest intensity (650±46 ms, F = 1.6, p = 0.24). Finally, RTs in the audio-visual condition did not differ from those during the visual-only condition, for any of the target intensities (e.g. at lowest intensity, 615±38 ms). We observed considerably animal by animal variability in RTs for a given condition (min 489 ms, max 773 ms for 0.015 cd/m^2^) and we performed an additional test to see whether this between-animal variably in RTs obscured potential difference between conditions. We therefore computed normalized RTs, defined by subtraction the average RT of each animal (computed across all conditions and intensities) from the individual conditions (Fig. 2A lower panel), which discounts for the (here irrelevant) animal by animal variability in average RT. However, this did not reveal a systematic difference between visual and audio-visual conditions (at least p>0.1 for all intensities).

Because studies with human subjects usually find significant reaction time benefits in comparable audio-visual tasks, we re-tested the animals after a prolonged period of training and acquaintance on the task (25 training days on the full task, 43 days in total). Psychophysical testing after this longer training epoch revealed significant benefits for both performance and reaction times (Fig. 2B). As before, performance increased with visual intensity and did not differ significantly between auditory (65±3%) and lowest intensity visual stimuli (69±3%, F(127,1) = 1.8, p = 0.2). However, performance levels were significantly enhanced for multisensory stimuli at the three lowest intensities (Fig. 2B upper panel). For example, at the lowest intensity audio-visual performance (79±2%) was significantly higher than visual performance (F = 16.8, p<0.01). RTs decreased with target intensity and RTs during the auditory condition were comparable to those for lowest intensity visual condition (auditory: 662±35 ms vs. visual: 626±31 ms, F = 2.4, p = 0.15). Importantly RTs were faster in the audio-visual compared to the visual condition, demonstrating a multisensory reaction time benefit. This effect was visible in the actual RT values (at lowest visual intensity 559±28 ms for audio-visual and 626±31 ms for visual conditions) and was even more pronounced after inter-subject normalization (Fig. 2B lower panel). For the normalized data this multisensory RT benefit was significant at the two lowest intensities in the normalized data (both p<0.05; Fig. 2B).

The behavioral responses revealed by the rats are in concordance with those known from human psychophysics where subjects exhibit better detection rates and faster reactions for audio-visual targets when presented as multisensory pair [22], [28], [34], [35], [36]. Such multisensory behavioral benefits were found over a range of experimental settings and also for animal species such as the cat [28], [37]. Studies on the detection of audio-visual targets have revealed that both targets need to be in close spatial and temporal proximity in order to produce behavioral benefits [28], [38]. These constraints are known as the spatial and temporal ‘rules’ of sensory integration [28], [36] and tests of such spatio-temporal constraints on multisensory integration could be easily implemented in future variants of the proposed task. This also opens the possibility to investigate multisensory integration properties in rodent models for diseases and to link these to known alterations in temporal integration windows known from dyslexic or autistic patients [39], [40]. In addition our data show that the rats behavior also seems to follow another basic rule of multisensory integration, the principle of inverse effectiveness [5], [28], [41]. This posits that behavioral benefits of multisensory stimuli are stronger under conditions where the individual unisensory stimuli provide little evidence or are little effective in driving the sensory systems. Indeed, the multisensory performance gain (computed as 100*(AV−V)/V) was largest at the lowest intensity and became progressively smaller for brighter visual stimuli (13.4±4%, 8.5±2% and 2±1% at 0.015, 0.06 and 0.5 cd/m^2^ respectively).

### Psychophysical test with a softer sound

**Figure 3:**
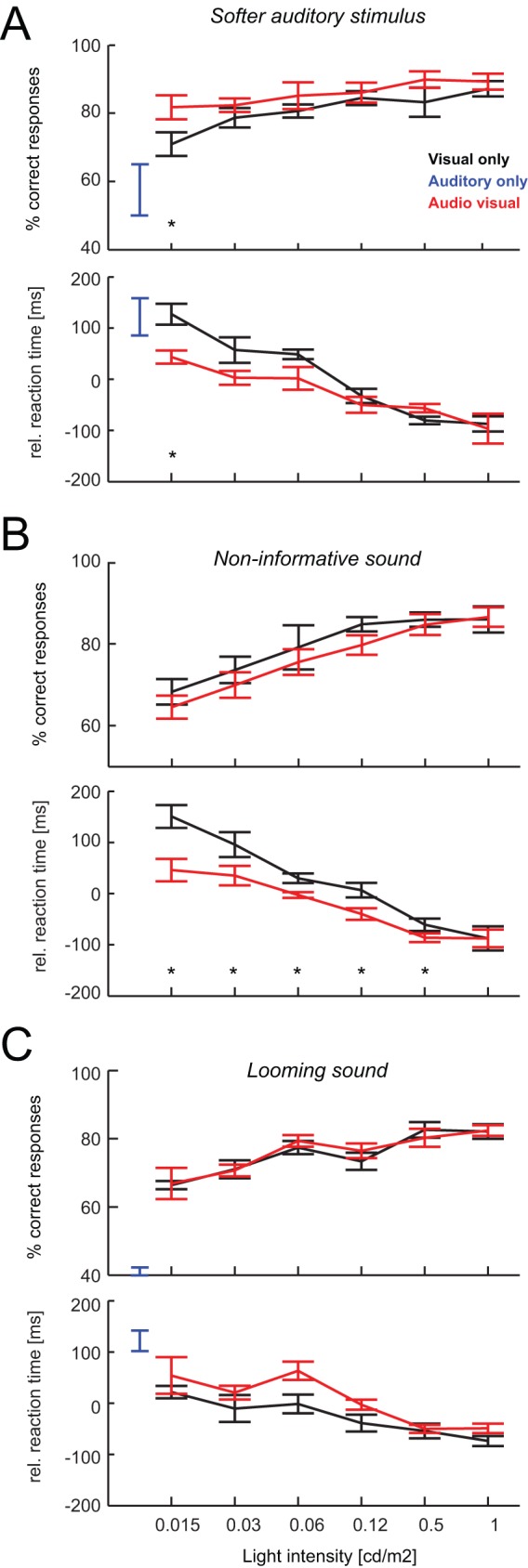
Behavioral performance – controls and additional paradigms. Behavioral performance (% correct responses, upper panel) and reaction times (lower panel) as in Figure 2 (n = 4 animals each). Stars indicate statistical significance (ANOVA p<0.05). A) Paradigm with a softer auditory stimulus (65 dB). B) Paradigm with a non-informative sound, hence no auditory performance level. C) Paradigm using looming sounds instead of white noise.

The above behavioral data were acquired using a paradigm that systematically manipulated the intensity of the visual target, while keeping sound intensity constant. In separate sessions (following more than 25 days of training) we tested a group of four animals using a softer acoustic stimulus (65 dB compared to 80 dB above). This confirmed several of the above findings, especially those of enhanced performance levels and faster RTs in the audio-visual compared to visual conditions at low visual stimulus intensities (Fig. 3A). Using the softer sound performance for auditory stimuli was considerably lower (58±7%) than for the lowest visual intensity (71±3%), although this did not reach significance due to day by day variability in auditory performance (F(63,1) = 3.1, p = 0.1). However, performance levels were significantly higher in the audio-visual compared to the visual condition at the lowest visual intensity (81±3% vs. 71±3%, F = 15, p<0.01). Reaction times also revealed a multisensory behavioral benefit as they were significantly shorter in the audio-visual compared to the visual condition at lowest visual intensity (580±50 ms vs. 664±62 ms, F = 11.6, p<0.05).

Comparing the behavioral data across conditions with louder and softer sounds (using data for only the same four animals tested in both paradigms) revealed that the softer sound induced only a minor change in behavioral performance. Detection performance in the auditory condition was 58±7% for the softer and 65±3% for the louder tone, reaction times 659±80 ms compared to 620±60 ms, and the magnitude of the multisensory performance benefit (computed as 100*(AV−V)/V at lowest visual intensity) 15±6% and 14±4% respectively. This shows the robustness of the facilitatory multisensory effect across a range of acoustic stimulus intensities and highlights that a chance in complementary auditory stimulus by 15 dB rendered unisensory responses only a little worse or slower.

### Psychophysical test with a non informative sound

Previous studies in humans have shown that perceptual detection and discrimination of sensory stimuli in one modality can also benefit from non-informative stimuli in another. For example, the detection of faint visual targets in a two-response forced choice task was enhanced when visual targets were accompanied by a sound that was not informative about the location of the visual target [26] (see also [42]). Multisensory benefits in this scenario do not reflect the integration of multisensory cues (which are only available in one modality) but likely reflect the enhancement of sensory processing by arousing or attention-increasing influences provided by the second modality. We implemented such conditions and performed a psychophysical test in which we made the acoustic stimulus non-informative about the lateralization of the visual stimulus (the sound appeared simultaneously on both sides). This test was done subsequent to those with the informative sound presented above.

We found that a non-informative sound had no significant impact on performance levels. While performance was lower for the audio-visual compared to the auditory condition across all visual intensities (e.g. at lowest intensity 64±3% vs. 68±3%), this difference did not reach statistical significance (p>0.05 for all; Fig. 3B). However, reaction times became faster in the audio-visual compared to the visual condition (e.g. lowest intensity: 569±67 ms vs. 674±58 ms; F(63,1) = 60, p<10^−3^). This shortening of RTs was significant for all but the highest visual intensity (at least p<0.05; Fig. 3B) demonstrating a clear behavioral benefit (faster reaction) at only a small cost in reduced performance level due to the non-informative sound. This finding resembles that obtained in the human study [26] and highlights the impact of both task-relevant and task-irrelevant stimuli on multisensory behavior in the rat. The facilitatory effect on reaction times shows that task irrelevant stimuli can have alerting and hence facilitatory influences on target perception both in rats and humans [23], [43].

While performance levels did not differ significantly between visual and audio-visual conditions in this paradigm a direct comparison of the behavioral data across paradigms with informative (80 dB) and non informative sounds hints upon a systematic difference. Multisensory performance was generally higher than unisensory performance for the informative sound, while it was generally lower for the non informative sound. For example (using data from only the 4 animals tested in both conditions) at the lowest visual intensity performance was 81±2% for the informative but only 65±3% for the non informative sound, suggesting that potential differences between unisensory and multisensory performance levels were masked by the small population size for this experiment.

### Psychophysical test with looming sounds

In a final behavioral test we probed whether looming sounds enhance the multisensory behavioral benefit that we found for white noise sounds. Looming sounds are complex sounds of great behavioral importance for animals as they for example reflect the approach of an object such as a predator. Because of this relevance it is thought that animals have evolved a bias for detecting and responding to looming sounds [44], [45], [46] and electrophysiological studies have revealed privileged integration of audio-visual looming signals [30], [47]. We speculated that replacing the white noise sound used in the previous tests by looming sounds (similarly lateralized) might possibly result in stronger multisensory benefits.

We tested a group of four animals that was previously naive to the looming sounds. In contrary to our expectation, we did not find any benefit of the acoustic stimulus on visual target detection (Fig. 3C). Performance levels for the auditory condition were below chance (41±2%) and did not differ between visual and audio-visual conditions for any intensity (e.g. lowest intensity: 67±5% vs. 66±1%, F(63,1) = 0.2, p = 0.9, at least p>0.05 for all other conditions). Similarly, RTs did not differ between visual and audio-visual conditions (e.g. lowest intensity: 610±69% vs. 580±47%, F(63,1) = 1.0, p = 0.33, at least p>0.05 for all other conditions). We interpret this lack of result as being due to the novelty of the sounds, which may have been distractive rather than useful for the task. In addition, and possibly more important, behaviorally relevant looming sounds for rodents may have a different acoustic structure than those sounds used here. We employed sounds similar to ones previously used in studies with monkeys [30], [45], [47] and humans [48], [49], but these may not have the same alerting effects on rats. To date there seems to be little knowledge about the general behavioral relevance of looming sounds in rodents, leaving the potential to develop more suitable acoustic looming stimuli for future studies.

## Conclusions

Our every day behavior takes great benefits from the fact that we have access to information provided by our different sensory modalities. The neural circuits and principles underlying multisensory integration are a timely topic of current research, but are often difficult to study in primate subjects. Rodents offer the promise to bridge some of the gaps between behavior and the underlying neural circuits and initial studies already highlighted the power of rodent models to understand the pathways underlying specific aspects of multisensory processing [17]. However, a larger set of multisensory behavioral protocols is required to elucidate the various aspects of multisensory processing and integration and their underlying neural networks, computational principles and pharmacological mechanisms. We demonstrate that rats exhibit multisensory behavioral benefits in an audio-visual detection task similar to other species, such as humans, primates and cats [28]. Together with other tasks where rodents perform multisensory temporal integration [20], speeded responses to multisensory targets [18] or visual- olfactory object recognition [21] our results pave the way for future studies exploring the rodent as model system to study the neural basis of multisensory perception. Such studies may not only provide crucial insights into the mechanisms underlying the perception of every day stimuli but also provide key insights about changes in multisensory integration in different disease states [38], [39], [40].

Several of our findings are noteworthy. Most importantly, we observed multisensory benefits for reaction times only after a longer (43 days in total) but not shorter (28 days) periods of training, while benefits for detection rates were already found after the shorter training period. This suggests that behavioral training of rats shapes their levels of perceptual performance and reaction times differently, with changes in reaction times requiring prolonged task acquaintance. This difference in the evolution of correct responses and reaction speed may have important implications for the interpretation of other studies that assess rodent behavior in a variety of paradigms. Reaction time enhancement is frequently used to quantify multisensory benefits in diverse behavioral conditions and has for example been studied in the context of aging [50], [51] or autism spectrum disorders [52]. Animal models for aging or disease related changes in multisensory processing will have to be carefully assessed against the differential expression of multisensory benefits for correct responses and reaction times with prolonged behavioral training. Recent work also highlighted multisensory benefits for the learning of perceptual tasks, showing that human subjects reach stable performance levels more quickly when performing a discrimination task in a multisensory rather than unisensory context [53], [54]. It will be interesting to see whether dissociations between changes in task performance level and reaction times with training proficiency occur also in humans, and whether the appearance of these multisensory benefits is related to task proficiency being acquired in a multisensory environment, or whether these occur also after unisensory training.

We further found that multisensory enhancement of reaction times occurred for both informative and non informative sounds while enhancement of correct detection rates occurred only for the informative sound. This highlights that potentially distinct neural networks or brain areas underlie speeded responses and the correctly lateralized detection of the target. While future studies are required to elucidate the underlying neural circuits using the proposed rat model, one may speculate that dissociations between reaction time and detection benefits rely on distinct subcortical (e.g. collicular) and cortical circuits. The colliculus has classically been associated with enhanced detection and speeded reactions [5] while cortical structures supposedly mediate more detailed and feature based multisensory integration [7], [11].

Our results also revealed audio-visual response benefits for both softer (65 dB) and louder (80 dB) sound levels. This shows that audio-visual interactions in the rat are robust across the range of sound intensities typically used in human studies [26], [48], [55]. Future studies may test the multisensory interactions more systematically as a function of sound level. Finally, we observed that an uninformative sound decreased multisensory compared to unisensory performance levels, while an informative sound resulted in the opposite. This decrease in performance however was not significant in our sample. More systematic behavioral tests using a larger population size may be required to assess the qualitative and quantitative differences between informative and non-informative sounds in altering performance levels. Altogether our results describe a versatile rat model for multisensory integration, highlight pitfalls with regard to the interpretation of reaction time and performance benefits, and provide multiple starting points for future work on the underlying neural substrate.
